# Proliferation of Acid-Secretory Cells in the Kidney during Adaptive Remodelling of the Collecting Duct

**DOI:** 10.1371/journal.pone.0025240

**Published:** 2011-10-24

**Authors:** Desa Welsh-Bacic, Marta Nowik, Brigitte Kaissling, Carsten A. Wagner

**Affiliations:** 1 Institute of Anatomy, University of Zurich, Zurich, Switzerland; 2 Institute of Physiology, University of Zurich, Zurich, Switzerland; 3 Zurich Center for Integrative Human Physiology, University of Zurich, Zurich, Switzerland; University of Houston, United States of America

## Abstract

The renal collecting duct adapts to changes in acid-base metabolism by remodelling and altering the relative number of acid or alkali secreting cells, a phenomenon termed plasticity. Acid secretory A intercalated cells (A-IC) express apical H^+^-ATPases and basolateral bicarbonate exchanger AE1 whereas bicarbonate secretory B intercalated cells (B-IC) express basolateral (and apical) H^+^-ATPases and the apical bicarbonate exchanger pendrin. Intercalated cells were thought to be terminally differentiated and unable to proliferate. However, a recent report in mouse kidney suggested that intercalated cells may proliferate and that this process is in part dependent on GDF-15. Here we extend these observations to rat kidney and provide a detailed analysis of regional differences and demonstrate that differentiated A-IC proliferate massively during adaptation to systemic acidosis. We used markers of proliferation (PCNA, Ki67, BrdU incorporation) and cell-specific markers for A-IC (AE1) and B-IC (pendrin). Induction of remodelling in rats with metabolic acidosis (with NH_4_Cl for 12 hrs, 4 and 7 days) or treatment with acetazolamide for 10 days resulted in a larger fraction of AE1 positive cells in the cortical collecting duct. A large number of AE1 expressing A-IC was labelled with proliferative markers in the cortical and outer medullary collecting duct whereas no labeling was found in B-IC. In addition, chronic acidosis also increased the rate of proliferation of principal collecting duct cells. The fact that both NH_4_Cl as well as acetazolamide stimulated proliferation suggests that systemic but not urinary pH triggers this response. Thus, during chronic acidosis proliferation of AE1 containing acid-secretory cells occurs and may contribute to the remodelling of the collecting duct or replace A-IC due to a shortened life span under these conditions.

## Introduction

The collecting duct is the major site of urinary acidification [1], a process that involves at least two subtypes of intercalated cells. Type A intercalated cells (A-IC) secrete protons into urine via a luminal H^+^-ATPase and express on the basolateral side the chloride/bicarbonate exchanger AE1 (Band3) [2], [3]. In contrast, non-type A intercalated cells are characterized by the apical expression of the chloride/bicarbonate exchanger pendrin [4], secrete bicarbonate into urine, and express luminal, basolateral or bipolar H^+^-ATPases [3]. Based on the localization of H^+^-ATPases some authors distinguish two subtypes of these intercalated cells, type B intercalated cells (with basolateral H^+^-ATPase) and non-A/non-B intercalated cells (luminal H^+^-ATPase) [5], [6].

During changes in systemic acid-base or electrolyte status, the collecting duct system (the connecting tubule (CNT), cortical collecting duct (CCD), outer and inner medullary collecting ducts (OMCD and IMCD) is remodelled and the relative number of the different subtypes of intercalated cells and segment specific cells (connecting tubule cells and principal collecting duct cells) as well as their morphology alter. Enhanced urinary acid excretion is accompanied by increased relative number of acid-secretory intercalated cells [7], [8]. Acid-loading of mice, rats or rabbits increases the number of intercalated cells that express luminal H^+^-ATPases and secrete protons [7], [8], [9], [10], [11], [12], [13]. Whether these cells were all type A intercalated cells remained open. Other studies, however, used more refined morphological criteria including electron microscopy or staining for AE1 as specific marker for type A intercalated cells [11], [12].

Intercalated cells were thought to be terminally differentiated and to lack the ability to further proliferate [14], [15], [16]. Remodelling of the collecting duct has therefore been thought to involve the interconversion of mature and fully differentiated type A and B intercalated cells, a process termed plasticity [14], [15]. In vitro and in vivo experiments provided evidence that hensin, a component of the extracellular matrix, may be involved and required for this adaptive process [14], [17], [18], [19].

Several lines of evidence support the novel concept that the many types of epithelial cells along the nephron retain or regain their ability to proliferate, both under normal conditions [20] as well as in response to different stimuli [21], [22], [23], [24], [25], [26]. Among these cells, also intercalated cells were noted to stain for markers of proliferation raising the possibility that regulated proliferation of intercalated cells may contribute to the adaptive remodelling of the collecting duct. Indeed proliferation of intercalated cells during acidosis has been demonstrated in mouse kidney and it was shown that GDF-15 may play a role in the early phase of this proliferative response [25]. Here we extended these observations and demonstrate that in rat kidney fully differentiated type A intercalated cells proliferate in response to systemic acidosis, whereas non-type A intercalated cells do not proliferate under these conditions. Regional differences along the nephron exist and functional data suggest that systemic but not urinary pH is relevant for triggering the proliferative response.

## Materials And Methods

### Animals

Male Wistar rats (120–150 g) (Janvier, Belgium) were used. Animals had free access to water and food. Sucrose, NH_4_Cl, NaCl, or acetazolamide were added to the drinking water as detailed below. Rats were treated in 2 series:

Series 1: Group 1: 2% sucrose for 12 hrs, 4 or 7 days (control); group 2: 0.28 M NH_4_Cl plus 2% sucrose for 12 hrs, 4 days, or 7 days.

Series 2: Group 1: 2% sucrose for 7 days (control); group 2: 0.28 M NH_4_Cl plus 2% sucrose for 4 or 7 days; group 3: 0.28 M NaCl plus 2% sucrose; group 4: 300 mg/l acetazolamide for 10 days. All animals in series 2 received BrdU injections (10 mg/kg body weight of 10 mg/ml 5-bromo-2′-deoxyuridine (BrdU) (Sigma-Aldrich, St. Louis, MO) dissolved in 0.9% NaCl s.c.) every 12 hrs for the last 4 days before fixation for immunohistochemistry. All groups consisted of at least 4 rats per time point and treatment for immunohistochemistry and 4 rats for harvesting kidneys for immunoblotting. All animals were kept in metabolic cages 48 hrs before sacrifice. Food and water intake and urine output were monitored by collecting urine over 24 hrs periods under light mineral oil. Before sacrifice, animals were anesthetized with isoflurane and arterial blood was taken from the tail artery for blood gas and electrolyte analysis. All experiments were performed according to Swiss Animal Welfare laws and were approved by the local veterinary authorities (Kantonales Veterinäramt Zürich)(Regulation von renalem Transport in der Ratte, protocol no 52/2004).

### Blood and urine analysis

Arterial blood was injected into a blood gas analyzer (Radiometer Copenhagen, ABL 505, Denmark) and the following values were determined: pH, HCO_3_^−^, pCO_2_, K^+^, Na^+^, Cl^−^. Urine pH was measured immediately. Ion chromatography (Metrohm ion chromatograph, Switzerland) was performed to obtain K^+^, Na^+^, Cl^−^ concentrations in urine samples. Urine creatinine was analyzed applying the Jaffé method [27], [28]. Urine ammonium concentration was determined according to the Berthelot Protocol [29]. Determination of titratable acids was done according to Chan [30].

### Immunoblotting

Animals were anaesthetized, perfused with PBS to remove all blood, and kidneys were rapidly harvested. Cortex and medulla were separated by hand-dissection under a stereo-microscope. After homogenization in an ice-cold K-HEPES buffer (200 mM mannitol/80 mM K-HEPES/41 mM KOH/pH 7.5) with pepstatin, leupeptin, K-EDTA, and PMSF as protease inhibitors, the samples were centrifuged at 100,000 x g for 1 h at 4°C, and the pellet was resuspended in K-HEPES buffer containing protease inhibitors. After measurement of the total protein concentration (Bio-Rad, Hercules, CA), 75 µg of crude membrane protein was solubilized in Laemmli sample buffer, and SDS-PAGE was performed on a 10% polyacrylamide gel. For immunoblotting, the proteins were transferred electrophoretically from unstained gels to polyvinylidene fluoride membranes (Immobilon-P, Millipore, Bedford, MA). After blocking with 5% milk powder for 1 h, the blots were incubated with the primary antibodies (rabbit anti-rat AQP2 (kind gift from J. Loffing, Univ. of Zurich, Switzerland), guinea-pig anti-mouse pendrin 1.10.000 [31], rabbit anti-mouse AE1 1∶3.000 [32], mouse monoclonal anti-actin (42 kD, Sigma, St. Louis, MO) 1∶5000) either for 2 h at room temperature or overnight at 4°C. After washing off the primary antibody and subsequent blocking, blots were incubated with the secondary antibodies coupled to horse radish peroxidase or alkaline phosphatase, respectively (Promega, Madison, Wisconsin, USA) for 1 h at room temperature. Antibody binding was detected with the enhanced chemiluminescence ECL kit (Amersham Pharmacia Biotech, UK) or the CDP star kit (Roche, Mannheim, Germany) before detection with Diana III Chemiluminescence detection system and quantified with the Aida Image Analyzer software (Raytest, Germany).

### Immunohistochemistry

#### Tissue fixation and preparation

Rats were anesthetized by an intraperitoneal injection of pentobarbital (100 mg/kg body weight) and fixed by vascular perfusion [33]. The fixative contained 3% paraformaldehyde (PFA), 0.01% glutaraldehyde (GA) and 0.5% picric acid, dissolved in a 3∶2 mixture of 0.1 M cacodylate buffer (pH 7.4, added with sucrose, final osmolality 300 mosmol/l) and 4% hydroxyl ethyl starch (HES; Fresenius Kabi, Bad Homburg, Germany) in 0.9% NaCl. The kidneys were fixed for 5 min, and then rinsed by vascular perfusion with 0.1 M cacodylate buffer for 5 min. The kidneys were removed from the animal, cut in two-four millimeter thick slices and immediately frozen in liquid propane cooled down to −196°C by liquid nitrogen. Frozen kidney slices were cut into 4 µm thick cryostat sections. Sections were rinsed 3×10 min in 50 mM NH_4_Cl/PBS in order to wash out glutaraldehyde fixative and to reduce background staining. In all protocols that included detection of PCNA or BrdU the cryostat sections were microwaved for 15 min in 0.01 M citrate buffer at pH 6.0. After pretreatment in 5% normal goat serum in PBS, the cryostat sections were incubated overnight in a humidified chamber at 4°C with the primary antibodies (see below), diluted in PBS-1% BSA. After incubation with primary antibodies, sections were rinsed three times with PBS and covered for 1 h at room temperature in the dark with the appropriate secondary antibodies coupled to FITC or Cy3. After rinsing with PBS, the sections were finally plated on coverslips with DAKO-Glycergel (Dakopatts) containing 2.5% 1,4-diazabicyclo (2.2.2.) octane (DABCO; Sigma, St. Louis, MO, USA) as a fading retardant, and studied on an epifluorescence microscope (Polyvar, Reichert-Jung). For nuclear staining, 4′,6-diamidino-2-phenylindole (DAPI; Sigma, St. Louis, MO, USA), diluted 1∶200, was added to the secondary antibodies. Double/triple labellings were performed using cocktails of mouse, rabbit, and guinea-pig primary antibodies, and of the respective secondary antibodies. No cross-reactivity between primary and secondary antibodies was observed by omitting primary antibodies.

All sections from different animal groups within one series were processed simultaneously with the same dilutions of primary and secondary antibodies.

#### Antibodies

The following primary antibodies were used at the dilutions given: rabbit anti-AE1 1∶200 [32], human anti-AQP2 (kindly provided by M. Knepper, USA) 1∶50.000, rabbit anti-calbindin-D28k (SWANT, Bellinzona, Switzerland) 1∶50.000, guinea pig anti-pendrin diluted 1∶100.000, mouse monoclonal anti-bromodeoxyuridine (BrdU) clone 3D4 (BD Biosciences Pharmingen, San Diego, CA, USA) 1∶300, mouse monoclonal anti-proliferating cell nuclear antigen (PCNA) (Dako Cytomation clone PC 10) 1∶400, and rabbit anti-Ki67 (Novacastra Laboratories, Newcastle, UK).

#### TUNEL assay

Apoptotic cells were stained by the use of the TUNEL (terminal deoxynucleotidyl transferase mediated dUTP Nick End Labeling) method (Apop-Tag®; Chemicon; VWR International Dietikon, Switzerland), which specifically labels the 3′-OH blunted ends of the fragmented DNA. The kit was used as recommended by the supplier.

#### Quantification of cells

To count cells, six sections from each animal for each set of incubations were analyzed. Basolateral AE1-stained cells were counted as type A intercalated cells, cells with apical staining for pendrin were counted as non-type A intercalated cells. Non-stained cells were identified as principal cells. In a separate set of incubations, AQP2 or calbindin-D28k were used to confirm principal cells. Single cells were positively identified by DAPI staining of nuclei. A total of 72 digital images (12 images for a section) were examined from each experimental animal with a total of 1200 counted cells/ animal.

### Statistics

All data are presented as means ± SEM. All data were tested for significance using the unpaired *t*-Test or one way ANOVA test, and only results with *p*<0.05 were considered statistically significant.

## Results

### Acute proliferation of collecting duct cells

Several protocols have been described that induce metabolic acidosis and remodelling of the collecting duct in mouse and rats including treatment with NH_4_Cl [34], [35], [36], [37]. In a first series of animals, NH_4_Cl was given to induce metabolic acidosis which was confirmed by blood gas analysis. Metabolic acidosis was evident in all groups given NH_4_Cl as shown by lower blood pH and bicarbonate levels (table 1).

**Table 1:** Arterial blood data group 1 (PCNA).

	control	12 hrs	4 days	7 days
		NH_4_Cl	NH_4_Cl	NH_4_Cl
**Blood**				
pH	7.46±0.03	7.29±0.01*	7.26±0.06*	7.37±0.05*
pCO_2_ (mmHg)	38.5±2.9	38.3±1.4	31.9±2.4*	36.8±2.5
HCO_3_^−^ (mM)	27.0±0.4	18.0±0.4*	14.8±2.3*	21.0±2.0*
Na^+^ (mM)	137.7±0.7	139.2±0.2	140.3±0.9	136.5±0.4
K^+^ (mM)	5.0±0.3	4.9±0.1	4.5±0.1	4.8±0.3
*Cl^−^ (mM)*	100.7±0.7	109.8±0.7*	113.0±4.1*	106.3±3.0*

Rats treated with NH_4_Cl in drinking water for 12 hrs, 4 or 7 days, n = 6 animals/ group. Data are given as mean ± SEM. * marks significant difference between control and treated group.

**Figure 1:**
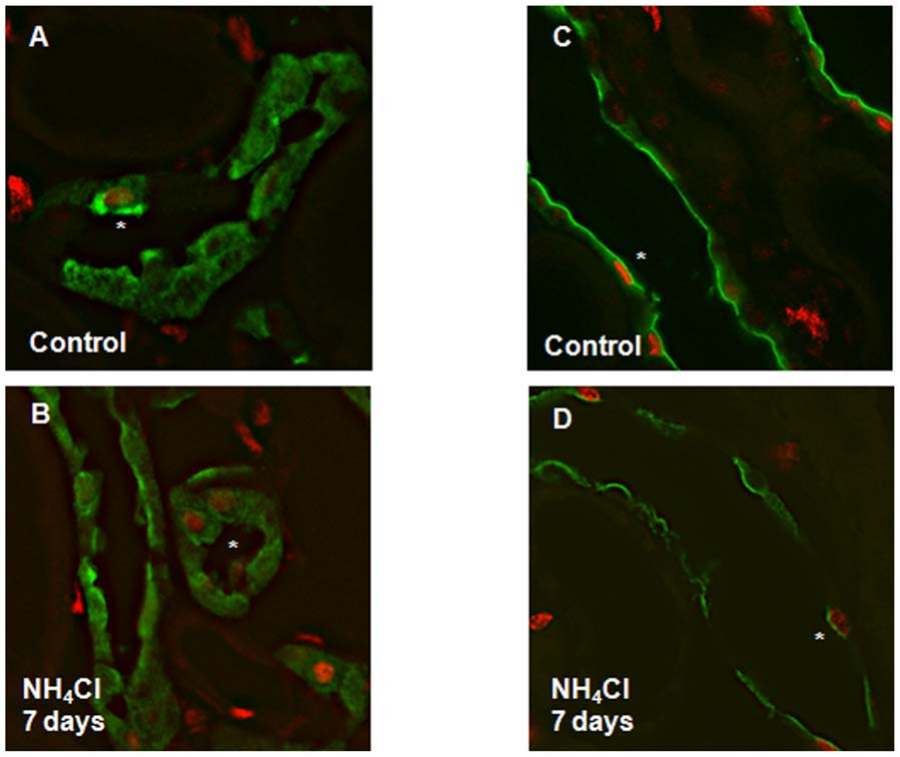
Proliferation of segment-specific cells after 7 days of acidosis in the CCD and OMCD. Animals were treated for 7 days with NH_4_Cl or were left untreated (controls). (**A,B**) Labelling of segment specific cells in the CCD with antibodies against calbindin D28K (green) in the CCD under control conditions and after 7 days of acidosis: PCNA staining (asterisk) was observed in calbindin D28k positive cells under control conditions and after 7 days of NH_4_Cl. (**C,D**) Segment-specific cells in the OMCD were identified by staining of AQP2 (green). Similarly to the CCD, some OMCD principal cells were positive for PCNA (asterisk) under control and acidotic conditions.

**Figure 2:**
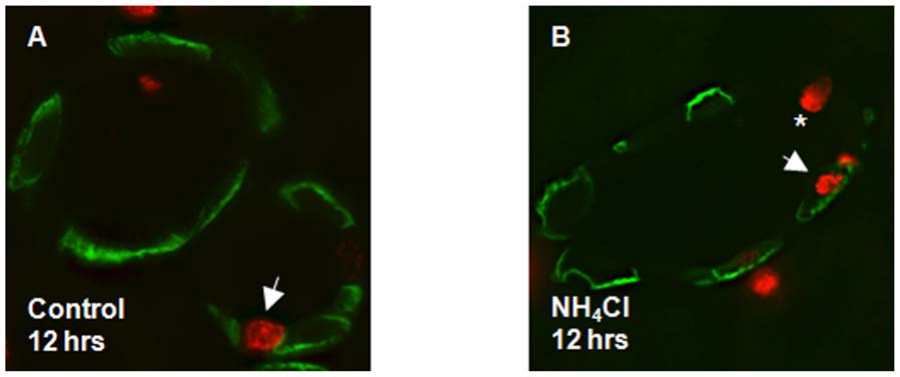
Early proliferation during induction and adaption to metabolic acidosis. Appearance of PCNA positive type A intercalated cells during acidosis. Kidney sections were stained with antibodies against either the type A intercalated cell specific AE1 protein (green), or the non-type A intercalated cell specific pendrin protein (red, asterisk), and antibodies against PCNA (red nuclei). (**A**) In the OMCD, PCNA labelling was seen in single cells not stained for AE1 under control conditions (arrow). (**B**) Co-staining of PCNA was seen in a few AE1 positive cells in the OMCD after 12 hrs NH_4_Cl-loading (arrow).

**Figure 3:**
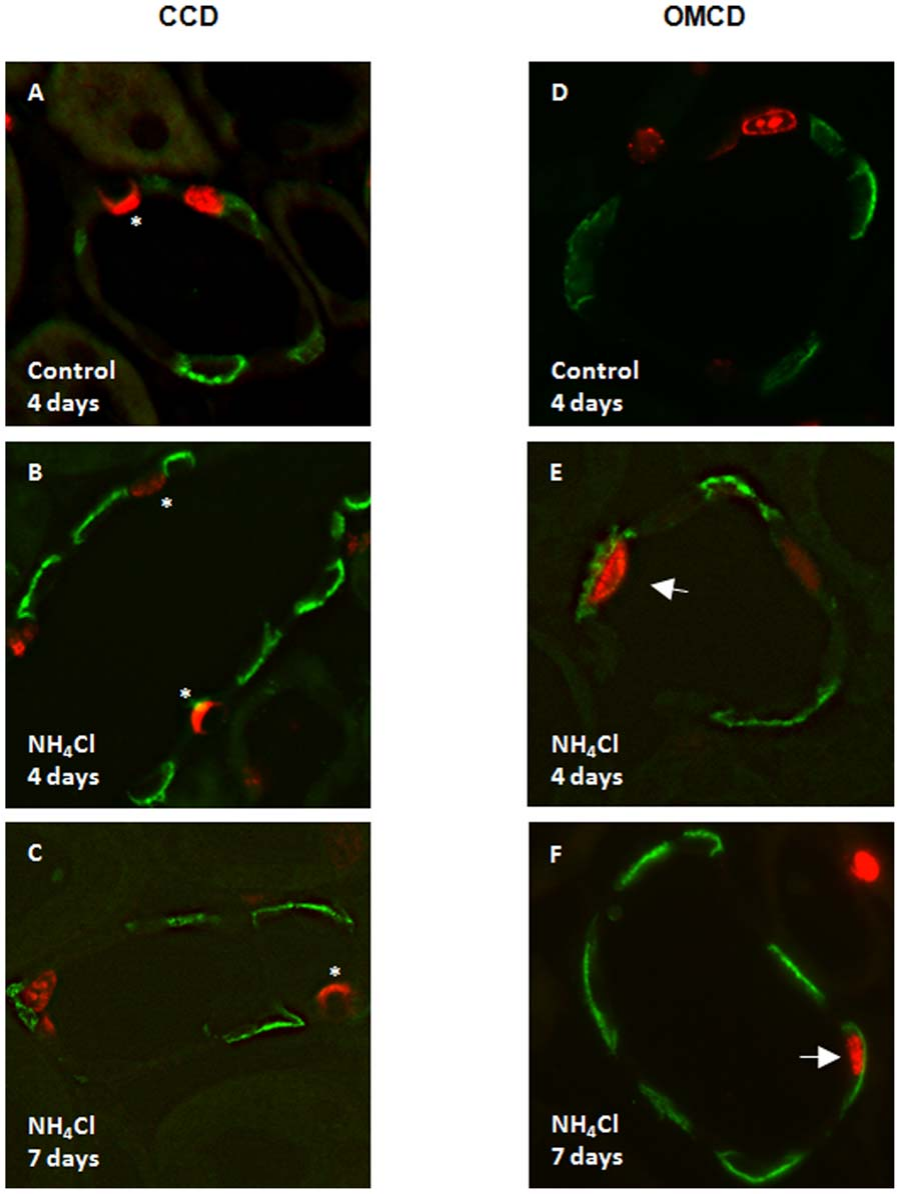
Proliferation of intercalated cells after 4 days of acidosis is observed in the OMCD but not CCD. Kidney sections were stained with antibodies against either the type A intercalated cell specific AE1 protein (green), or the non-type A intercalated cell specific pendrin protein (red, asterisk), and antibodies against PCNA (red nuclei). Animals were treated for 4 days with NH_4_Cl or were left untreated (controls). (**A–C**) Colocalization of PCNA and AE1 in some cells in the CCD after 4 or 7 days of NH_4_Cl treatment: Pendrin positive cells (*) in the CCD did not show PCNA labelling at any time point. (**D–F**) In the OMCD PCNA labelling was found under control conditions in AE1 negative cells (D) whereas colocalization (arrows) with AE1 could be observed after 4 and 7 days of metabolic acidosis (E,F).

Acutely proliferating cells express the PCNA and Ki67 antigens in their nuclei and can be identified based on immunohistochemistry [26], [38]. Antibodies against PCNA or Ki67 were combined with antibodies directed selectively against cell-specific markers of type A (AE1) [2] and non-type A (pendrin) [4] intercalated cells, respectively, as well as against segment-specific cells (AQP2 and calbindin D28k) [39]. We did not observe any colocalization in the same cell of these cell-specific markers in any segment of the collecting duct under all conditions tested (data no shown) in agreement with previous reports [2], [5], [39], [40], [41]. PCNA and Ki67 labelling showed identical results (data not shown) and we therefore used in the following only PCNA for a more detailed analysis. In kidneys from control animals, a small number of cells in the CNT, CCD, and OMCD stained positive for PCNA and all these cells were positive for AQP2 or calbindin D28k demonstrating a low rate of proliferation of segment-specific cells in normal rat kidney (table 2, figure 1). No colocalization with PCNA was detected in cell stained for pendrin or AE1 under control conditions (figures 2 and 3).

**Table 2:** PCNA labeled cells.

	AE1+ (%)	AE1+ /PCNA+ (%)	PDS+ (%)	PDS+ /PCN+ (%)	AQP2+ (%)	AQP2+ PCNA+ (%)	Total cell number
**CNT**							
Control #1	25.4±0.8	0±0	6.5±0.8	0±0	68.2±1.6	1.9±0.3	1849
NH_4_Cl 12 hrs	25.5±1.1	0±0	4.6±0.4	0±0	70.0±0.8	1.9±0.3	2457
NH_4_Cl 4d	25.9±0.8	0±0	6.5±0.5	0±0	67.6±1.3	2.0±0.2	1997
Control #2	26.5±0.4	0±0	5.5±1.1	0±0	68.0±1.3	2.0±0.2	1927
NH_4_Cl 7d	27.1±0.4	0±0	5.6±0.3	0±0	67.3±0.6	1.8±0.1	1924
**CCD**							
Control #1	12.4±0.6	0±0	17.3±1.0	0±0	70.3±1.3	1.8±0.7	1114
NH_4_Cl 12 hrs	13.0±0.4	0±0	17.1±0.7	0±0	70.0±0.5	2.5±0.4	1516
NH_4_Cl 4d	11.4±0.3	0±0	16.2±0.8	0±0	72.4±0.8	2.1±0.2	1213
Control #2	12.6±0.7	0±0	17.4±0.2	0±0	70.0±0.7	2.6±0.2	1152
NH_4_Cl 7d	14.6±0.8*	3.5±0.9*	11.1±0.4*	0±0	74.3±0.8	0.8±0.1*	1176
**OMCD**							
Control #1	35.8±1.3	0±0	0±0	0±0	64.2±1.3	1.6±0.5	566
NH_4_Cl 12 hrs	37.3±0.5	3.2±0.5*	0±0	0±0	62.7±0.5	2.4±0.6	811
NH_4_Cl 4d	37.7±1.1*	3.8±1.9*	0±0	0±0	62.3±1.2*	2.6±0.7	623
Control #2	35.5±1.0	0±0	0±0	0±0	64.1±1.0	1.9±0.3	582
NH_4_Cl 7d	37.9±1.0 *	2.5±0.7*	0±0	0±0	62.1±1.0*	2.4±0.5	614

Experiments were carried out in two separate series (#1 and #2) with the respective control groups. Data are given as mean ± SEM. * marks significant difference between control and treated group.

**Figure 4:**
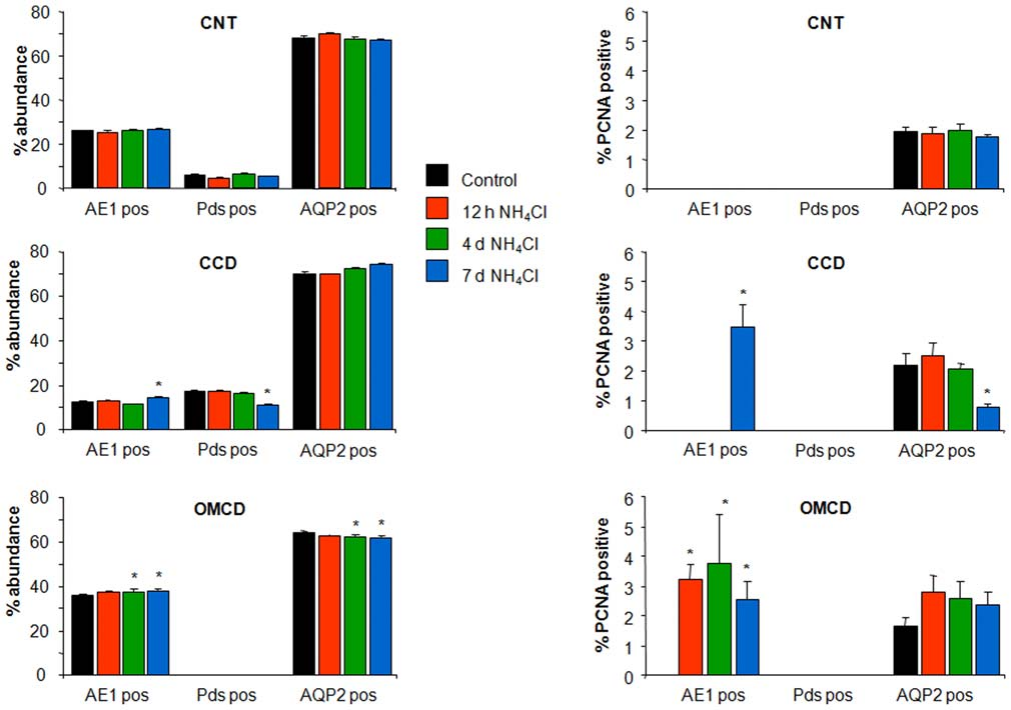
Remodelling and PCNA positive cell counts. (**A–C**) Summary of cells counts assessing the relative abundance of AE1, pendrin or AQP2 positive cells in the CNT, CCD, and OMCD of rats treated for 12 hrs, 4 or 7 days with NH_4_Cl in the drinking water. (**D–F**) Percentage of AE1, pendrin, or AQP2 positive cells that were also stained for PCNA under the different conditions. * marks significant difference compared to control.

Treatment of rats with NH_4_Cl caused remodelling of the collecting duct after 7 days as demonstrated by a small but significant increase in AE1 positive cells in the CCD from 12.6±0.7% to 14.6±0.8% and a concomitant reduction in the relative number of pendrin positive cell from 17.4±0.2% to 11.1±0.4 (table 2, figure 4). Also in the OMCD, the relative abundance of AE1 positive cells increased after 4 and 7 days of NH_4_Cl with a parallel reduction in AQP2 positive cells. No apparent changes in the relative cell numbers for AE1, pendrin or AQP2 positive cells could be found at any time point in the CNT. Hence, under the present experimental conditions remodelling in rat kidney may primarily occur in the CCD and OMCD with an earlier appearance in the OMCD. Similarly, after 7 days, a subset of AE1 positive cells in the CCD stained for PCNA, whereas in the OMCD colocalization of PCNA and AE1 was found as early as 12 hrs after induction of metabolic acidosis indicating that differentiated type A intercalated cells were undergoing proliferation (table 2, figures 2 and 4). The number of PCNA positive segment-specific cells did not differ between different treatment groups with one exception. The number of PCNA positive cells was lower after 7 days of NH_4_Cl treatment in the OMCD (figure 4, table 2).

### Cumulative rate of proliferation is increased during metabolic acidosis

PCNA and Ki67 mark only cells that acutely proliferate [26], [38]. In order to assess the rate of proliferation over a longer period of treatment, we used a second group of animals. Rats were injected every 12 hours with BrdU that is incorporated into the DNA of proliferating cells. In this second group of animals we also tested if loading with equimolar amounts of NaCl affected the rate of proliferation as chloride has been shown to affect the function and morphology of intercalated cells [31], [42], [43], [44]. We also included another group of rats, treated with the carbonic anhydrase inhibitor acetazolamide which induces a mild metabolic acidosis with alkaline urine and also causes remodelling of the collecting duct [13]. To further control the effects of the different treatments, animals were placed in metabolic cages for urine analysis, and kidneys were used to assess the regulated expression of cell specific markers (AE1, pendrin, AQP2) separately in cortex and medulla by immunoblotting.

**Figure 5:**
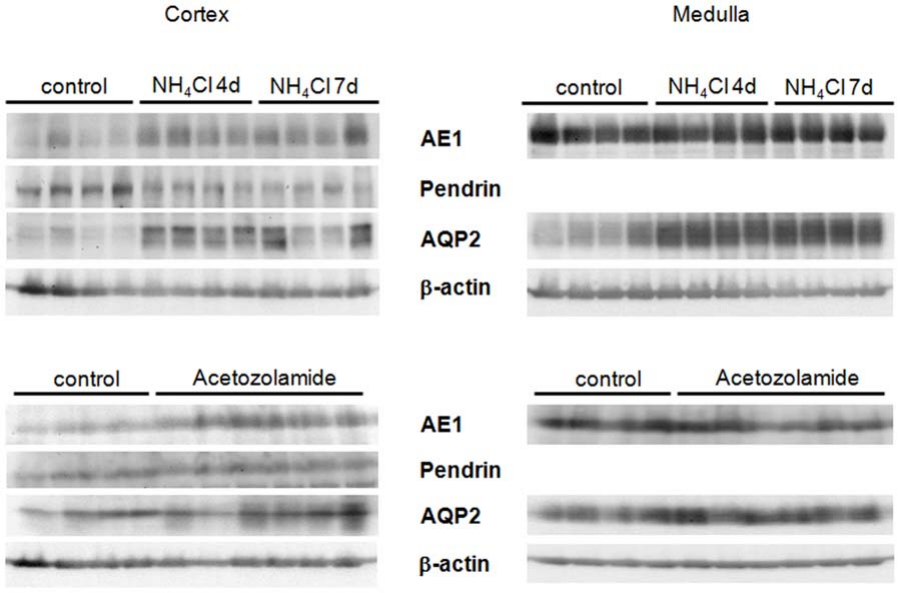
Regulated expression of AE1, pendrin, and AQP2 during different treatments. Immunoblotting of total membranes prepared from cortex and medulla demonstrated regulation of AE1, pendrin and AQP2 in the different treatment groups. N = 4 animals/ group.

Acid-loading with NH_4_Cl induced hyperchloremic metabolic acidosis (table 3) whereas NaCl-loading did not affect systemic acid-base status. Acetazolamide induced a very mild and compensated metabolic acidosis as evident from lower arterial bicarbonate levels (table 3). NH_4_Cl treatment stimulated urinary acidification and increased urinary acid excretion, whereas acetazolamide caused more alkaline urine and reduced urinary net acid excretion as expected. Immunoblotting showed increased abundance of AE1 and AQP2 in cortex and medulla after 4 and 7 days of NH_4_Cl-loading (Figure 5). Pendrin expression was reduced under these conditions. NaCl-loading also enhanced AE1 and AQP2 expression in cortex and medulla, whereas pendrin expression was decreased (data not shown). Acetazolamide treatment increased AE1 in cortex but not in medulla, pendrin expression remained unaltered, and AQP2 abundance slightly increased in cortex and medulla (Figure 5).

**Table 3:** Arterial blood and urine data group 2 (BrdU).

	control	NH_4_Cl	NH_4_Cl	NaCl	Acetazol
		4 days	7 days	7 days	10 days
Body weight at start (g)	127.6±1.7	128.9±3.1	126.6±2.0	134.1±2.2	134.2±1.4
Body weight at the end (g)	174.0±4.3	154.9 ±3.9	148.8±4.8	157.9±5.4	170.2±2.8
**Blood**					
pH	7.43±0.01	7.29±0.06*	7.37±0.06*	7.46±0.01	7.40±0.01
pCO_2_ (mmHg)	38.6±1.3	29.4±1.1*	31.1±2.4*	39.5±1.1	37.4±1.2
HCO_3_^−^ (mM)	25.5±0.5	14.1±1.9*	18.1±2.0*	27.7±1.3	22.8±0.4*
Na^+^ (mM)	136.0±0.7	135.6±1.7	136.0±1.4	143.5±2.7*	137.3±0.3
K^+^ (mM)	4.8±0.2	5.3±0.3	5.2±0.4	4.0±0.1*	4.3±0.1*
Cl^−^ (mM)	103.2±0.4	117.2±2.3*	111.0±3.4*	106.8±2.0*	105.8±0.8*
**Urine**					
pH	6.86±0.08	5.93±0.15*	5.99±0.13*	6.41±0.07	7.27±0.03*
Volume (ml/ 24 hrs)	28.8±3.4	10.0± 1.4*	9.5±1.0*	106.1±16.3*	19.4±1.5*
Creatinine (mg/dl)	15.5±3.1	25.3±2.7*	44.0±8.8*	3.3±0.4*	15.8±0.9
NH_4_^+^/ Creatinine (mM/mg/dl)	0.7±0.1	8.4±0.7*	8.4±0.8*	0.8±0.1	1.3±0.1*
Na^+^/ Creatinine (mM/mg/dl)	1.5±0.6	1.6±0.2	2.8±0.5	133.2±22.9*	3.5±0.3*
K^+^/ Creatinine (mM/mg/dl)	2.1±0.8	4.0±0.5	4.8±0.6	4.8±0.7	7.7±0.5*
Cl^−^/ Creatinine (mM/mg/dl)	2.0±0.7	11.1±0.6*	16.2±0.4*	131.9±21.3*	4.8±0.6
TA mmol/l	4.2±1.4	63.6±11.1*	63.9±7.3*	−0.5±1.0*	−42.1±3.1*
NAE mmol/l	14.5±1.9	353.0±18.1*	365.2±12.6	2.1±1.1	−20.5±4.7*
*NAE/Creatinine* *(mM/mg/dl)*	0.8±0.1	10.4±1.0*	10.1±1.0*	0.8±0.4	−1.4±0.3*

Rats treated with NH4Cl for 4 or 7 days, NaCl for 7 days, or acetazolamide for 10 days. Blood was taken under anesthesia from tail arteries, urine was collected over 24 hrs in metabolic cages. N = 10 animals/ group, * marks significant difference between control and treated group.

Values are means ± SEM, TA titratable acids, NAE net acid excretion.

**Figure 6:**
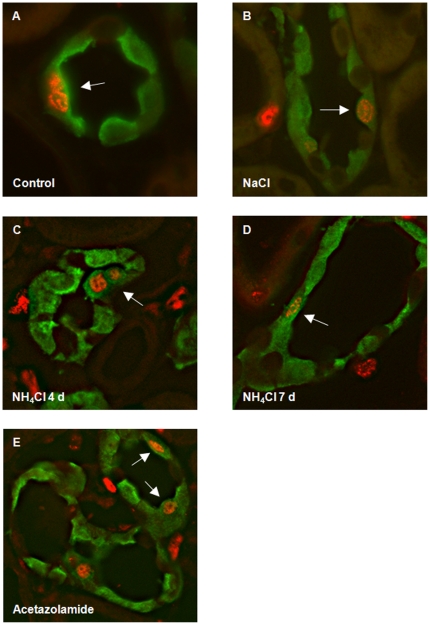
Proliferation of segment specific cells in the connecting tubule (CNT). Kidney sections were stained with antibodies against calbindin D28k (green), a marker of segment-specific cells in the CNT, and against BrdU (red). Colocalization of both stainings was observed in cells of animal groups in the CNT (arrows).

**Figure 7:**
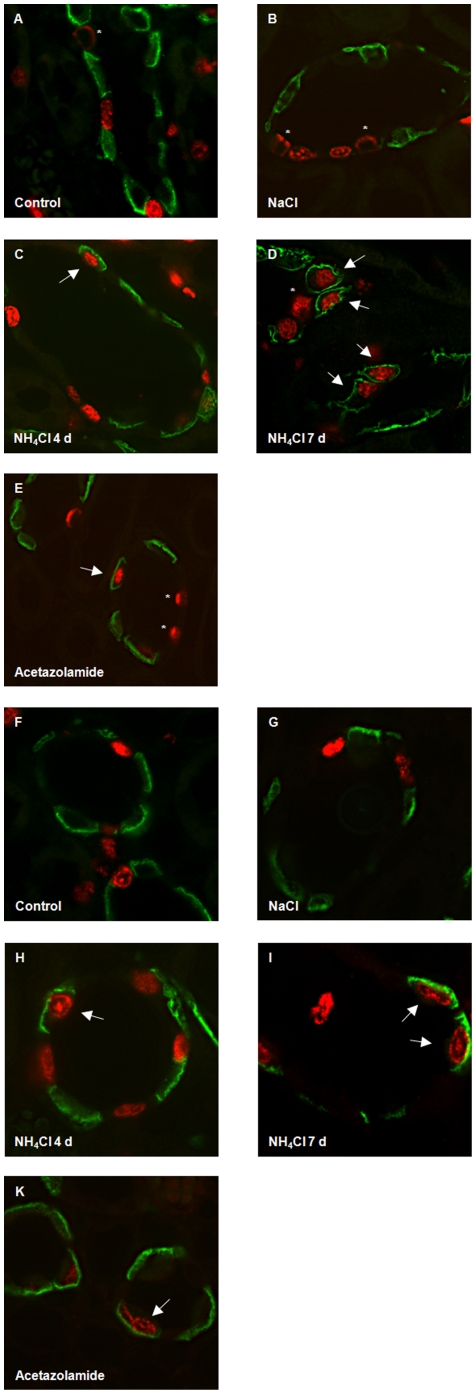
Proliferation of type A intercalated cells during metabolic acidosis. Kidney sections were stained with antibodies against AE1 (green), pendrin (red, asterisk), and BrdU (red nuclei). (**A–E**) Localization of BrdU positive cells in the CCD of control rats (A) or animals on NaCl for 7 days (B), NH_4_Cl for 4 or 7 days (C,D), or receiving acetazolamide for 10 days (E). BrdU colocalized with AE1 in NH_4_Cl or acetazolamide treated rats (arrows). (**F–K**) Colocalization of BrdU and AE1 staining (arrows) in OMCD intercalated cells in rats treated with NH_4_Cl (H, I) or acetazolamide (K) but not in control (F) or NaCl treated (G).

**Figure 8:**
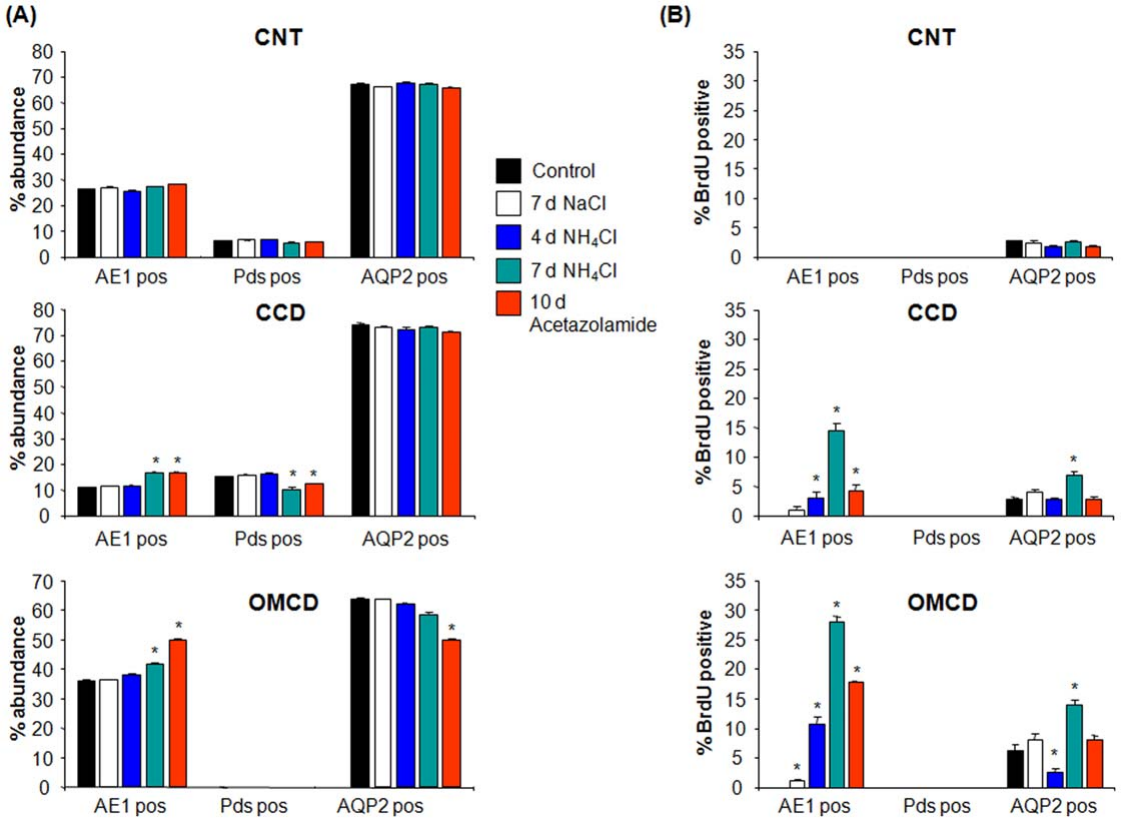
Remodelling and BrdU positive cell counts. (**A**) Summary of cells counts assessing the relative abundance of AE1, pendrin or AQP2 positive cells in the CNT, CCD, and OMCD of rats treated for 4 or 7 days with NH_4_Cl, 7 days with NaCl, or 10 days of acetazolamide in the drinking water. (**B**) Percentage of AE1, pendrin, or AQP2 positive cells that were stained for BrdU and the respective cell marker under the different conditions. * marks significant difference compared to control.

Immunohistochemistry detecting BrdU-labelled cells that had undergone proliferation in combination with cell-type specific markers showed no colocalization of BrdU with AE1 or pendrin in the connecting tubule (CNT) in all groups tested (Figure 6). In contrast, in the CCD and OMCD, NH_4_Cl-loading induced a time-dependent increase in proliferation with many type A intercalated cells having incorporated BrdU after 7 days (Figures 7 and 8, table 4). In the OMCD, 10.8±1.1% of all cells were positive for AE1 and BrdU, corresponding to about 25% of all intercalated had incorporated BrdU during 4 days of NH_4_Cl-loading. This number increased to 28.1±0.9% of all cells being positive for AE1 and BrdU after 7 days of NH_4_Cl-loading (or almost 80% of all OMCD intercalated cells)(table 4). Also acetazolamide induced BrdU labelling of about 50% of all type A intercalated cells. NaCl loading was without effect on type A intercalated cells. We did not detect colocalization of pendrin staining and BrdU labelling under any condition. Thus, chronic NH_4_Cl loading or treatment with acetazolamide are associated with significant rates of proliferation of type A intercalated cells in CCD and OMCD. We also searched for mitotic figures but detected only very few that did not allow further statistic analysis (data not shown).

**Table 4:** BrdU labeled cells.

	AE1 pos (%)	AE1 pos + BrdU pos (%)	PDS pos (%)	PDS pos + BrdU pos (%)	AQP2 pos (%)	AQP2 pos BrdU pos (%)	Total cell number
**CNT**							
Control	26.2±0.2	0±0	6.3±0.1	0±0	67.3±0.3	2.7±0.2	3262
NaCl	27.1±0.2	0±0	6.6±0.1	0±0	66.4±0.1	2.6±0.3	2570
NH_4_Cl 4d	25.8±0.3	0±0	6.6±0.2	0±0	67.5±0.5	1.9±0.2	3365
NH_4_Cl 7d	27.2±0.3	0±0	5.6±0.2	0±0	67.2±0.3	2.6±0.3	2606
Acetazol	28.2±0.2	0±0	6.0±0.2	0±0	65.8±0.3	1.9±0.1	2559
**CCD**							
Control	10.9±0.4	0±0	15.1±0.5	0±0	74.0±0.9	2.8±0.3	2049
NaCl	11.4±0.2	1.0±0.7	15.6±0.3	0±0	73.0±0.5	4.1±0.4	1754
NH_4_Cl 4d	11.5±0.3	3.0±1.2*	16.1±0.6	0±0	72.4±1.0	2.9±0.2	2157
NH_4_Cl 7d	16.6±0.4*	14.5±1.3*	10.1±0.2*	0±0	73.3±0.4	7.0±0.5*	2042
Acetazol	16.5±0.4*	4.3±1.3*	12.3±0.2*	0±0	71.2±0.4	2.9±0.4	1757
**OMCD**							
Control	36.2±0.5	0±0	0±0	0±0	63.8±0.5	6.3±0.9	1065
NaCl	36.3±0.3	1.2±0.1*	0±0	0±0	63.7±0.3	8.1±1.0	914
NH_4_Cl 4d	38.0±0.4	10.8±1.1*	0±0	0±0	62.0±0.4	2.6±0.6*	1077
NH_4_Cl 7d	41.6±0.7*	28.1±0.9*	0±0	0±0	58.4±0.7	14.1±0.8*	1288
Acetazol	50.1±0.4*	17.9±0.1*	0±0	0±0	49.9±0.4*	8.0±0.8	901

Data are given as mean ± SEM.

*marks significant difference between control and treated group.

**Figure 9:**
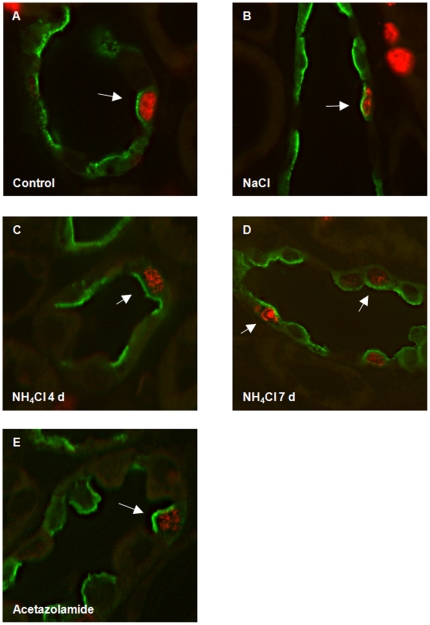
Proliferation of segment-specific cells in the outer medullary collecting duct (OMCD). Kidney sections were stained with antibodies against the AQP2 water channel (green), a marker of segment-specific cells, and against BrdU (red). Colocalization of both stainings was observed in cells of animal groups in the OMCD (arrows).

Few AQP2 or calbindin D28k stained segment-specific cells were positive for BrdU under control conditions indicating a low rate of proliferation of CNT, CCD, and OMCD segment-specific cells under basal conditions as described previously in mouse [21]. Chronic NH_4_Cl application (7 days) increased BrdU labelling of CCD and OMCD segment-specific cells (Figures 5, 7, and 9, table 4). Acetazolamide and NaCl application was without detectable effect on BrdU incorporation.

Detailed cell counting of type A intercalated cells, non-type A intercalated cells, and segment-specific cells demonstrated remodelling in the CCD and OMCD of rats treated with NH_4_Cl for 7 days resulting in an increased relative abundance of type A intercalated cells (figure 6, table 4) similar to the first series of animals. In addition, acetazolamide treatment also increased the relative number of type A intercalated cells in the CCD and OMCD at the expense of non-type A intercalated cells in the CCD and segment-specific cells in the OMCD, respectively (figure 6, table 4. These observations are consistent with previous reports using similar treatments [11], [13].

## Discussion

Remodelling of various nephron segments has been described in different genetically altered mouse models, disease models, or various physiological conditions [11], [13], [19], [21], [22], [23], [24], [45], [46], [47], [48], [49], [50]. Remodelling often contributes to the compensation of loss of function or the adaptive increase in transport capacity to match altered physiological conditions. Hypertrophy of cells, proliferation of cells, or removal of cells through apoptosis can often be observed during remodelling and may contribute to adaptation to various degrees. During metabolic acidosis, extensive remodelling of the collecting duct has been described including hypertrophy of intercalated cells and an increase in the relative cell number of acid-secretory type A intercalated cells over principal cells [11], [12], [31], [51]. Several observations may explain remodelling during metabolic acidosis. Interconversion of type A and non-type A intercalated cells has been described under in vitro conditions of acute acidosis such as in isolated perfused rabbit CCD [52] or in cell culture [16], [17], [53]. A role for hensin, an extracellular matrix protein, and β1 integrin in this process has been proposed [14], [19]. More recently, mouse models deficient for hensin or β1 integrin have been described that develop distal renal tubular acidosis and lack normal type A intercalated cells [18]. Thus, hensin and β1 integrin may be involved in the normal development of the collecting duct and differentiation of intercalated cells similarly to the transcription factors such as Foxi1 and CP2L1 or the signalling molecules GDF15 or Notch [25], [54], [55], [56]. However, the role of hensin, β1 integrin, Foxi1, CP2L1 and the Notch pathway in the adaptive remodelling of the collecting duct to acid-loading has not been determined in vivo to date. Mice lacking GDF-15 show a reduced adaptation to acute acid-loading with more pronounced acidosis and a lower number of intercalated cells proliferating suggesting that proliferation may contribute to the early phase of adaptation (e.g. 1–3 days) but not to the chronic phase [25].

Here we describe that cell proliferation of type A intercalated cells precedes and parallels remodelling in the CCD and OMCD of acidotic rats. Strikingly, no proliferation and no remodelling were observed in the CNT. Increased proliferation was observed as early as 12 hours after induction of acidosis and thereby preceded the remodelling of the collecting duct. During seven days of NH_4_Cl-loading about 15% of type A intercalated cells in the CCD and about one third of all intercalated cells in the outer medullary collecting duct had proliferated as indicated by BrdU incorporation. However, BrdU detects only cells that had proliferated but does not allow identifying the precursor cells. In contrast, the costaining of Ki67 or PCNA with AE1 or pendrin allowed the detection of fully differentiated intercalated cells during proliferation. PCNA (proliferating cell nuclear antigen) participates in DNA synthesis and is therefore detected only during the S-phase of the cell cycle [57], [58]. Ki-67 can be detected during all active phases of the cell cycle (G_1_, S, G_2_, and mitosis), but is not detected in resting cells (G_0_ phase) [59]. Thus, the detection of AE1 positive cells expressing also PCNA or Ki-67 identified without doubt these cells as fully differentiated and proliferating cells.

**Figure 10:**
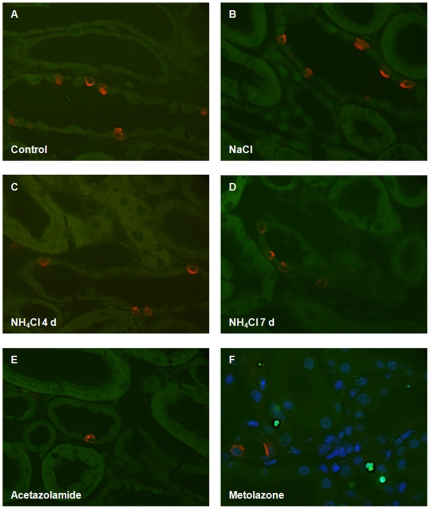
No detectable apoptosis of pendrin positive cells during metabolic acidosis. (**A–E**) Tissue sections were stained for pendrin (red) and TUNEL labelling (green) was performed to detect apoptotic cells. Under all conditions tested no apoptotic cells could be detected in the CNT, CCD or OMCD. (**F**) Apoptotic cells (green) were detected in kidneys from rats treated with the diuretic metolazone inducing apoptosis as described previously [50]. Cell nuclei were also stained with DAPI (blue).

Under the same conditions we did not find BrdU incorporation or colocalization of Ki67 or PCNA with pendrin suggesting that non-type A intercalated cells did not proliferate during acid-loading consistent with the reduction in the relative abundance of pendrin positive cells. Wehrli et al, however, has shown BrdU incorporation in pendrin positive intercalated cells in mouse kidney suggesting that also these cells may be capable of proliferation [21]. AQP2 positive cells showed under basal conditions as well as during the different treatments always BrdU incorporation and staining for PCNA or Ki67 confirming a previous report that showed also constant proliferation of segment-specific cells [21]. Our results do not indicate if acidosis increases the total number of acid-secretory cells or rather shifts the relative abundance at the expense of the other cell types. Removal of pendrin expressing intercalated cells from the medulla during kidney development by apoptosis has been described [60], [61]. We tested for the occurrence of apoptosis using the TUNEL assay but could not detect any positive intercalated cells whereas distal convoluted cells in thiazide treated rats stained positive as reported previously (figure 10) [50].

The signal for proliferation is most likely systemic acidosis but not acidic urine since we could observe a similar proliferative response with NH_4_Cl-loading and acetazolamide treatment. The first stimulates urinary acidification whereas the latter is associated with a more alkaline urine due to bicarbonate losses. Similarly, Bagnis et al had observed an increased abundance of type A intercalated in rat kidney after chronic treatment with acetazolamide [13]. However, the signal(s) stimulating type A intercalated cell proliferation and collecting duct remodelling remains to be determined.

In summary, we demonstrate that fully differentiated type A intercalated maintain or regain their ability to proliferate during adaptation to systemic acid-loading. The time course suggests that this proliferation participates in the remodelling of the collecting duct or may contribute to the replacement of type A intercalated cells.
